# Susceptibility to audio signals during autonomous driving

**DOI:** 10.1371/journal.pone.0201963

**Published:** 2018-08-13

**Authors:** Remo M. A. van der Heiden, Christian P. Janssen, Stella F. Donker, Lotte E. S. Hardeman, Keri Mans, J. Leon Kenemans

**Affiliations:** Experimental Psychology & Helmholtz Institute, Utrecht University, Utrecht, The Netherlands; Chongqing University, CHINA

## Abstract

We investigate how susceptible human drivers are to auditory signals in three situations: when stationary, when driving, or when being driven by an autonomous vehicle. Previous research has shown that human susceptibility is reduced when driving compared to when being stationary. However, it is not known how susceptible humans are under autonomous driving conditions. At the same time, good susceptibility is crucial under autonomous driving conditions, as such systems might use auditory signals to communicate a transition of control from the automated vehicle to the human driver. We measured susceptibility using a three-stimulus auditory oddball paradigm while participants experienced three driving conditions: stationary, autonomous, or driving. We studied susceptibility through the frontal P3 (fP3) Electroencephalography Event-Related Potential response (EEG ERP response). Results show that the fP3 component is reduced in autonomous compared to stationary conditions, but not as strongly as when participants drove themselves. In addition, the fP3 component is further reduced when the oddball task does not require a response (i.e., in a passive condition, versus active). The implication is that, even in a relatively simple autonomous driving scenario, people’s susceptibility of auditory signals is not as high as would be beneficial for responding to auditory stimuli.

## Introduction

Human driving involves various tasks, ranging from control (or operational) tasks such as basic vehicle control, to maneuvering (or tactical) tasks such as a lane change, to strategic tasks such as navigation [1]. With the advent of semi-autonomous cars and other automated vehicles, the role of the human driver changes. Some tasks might be automated partially, or even fully, and left to the system to control, whereas other tasks might still remain with the driver. As automation increases, the role of the human changes more from an active driver to that of a system monitor (e.g., [2]).

What the human driver needs to monitor or act on, depends on the degree of automation, or autonomy, of the vehicle. To classify the role division between human and system, the Society of Automotive Engineering [3] has distinguished six levels of automation. These range from level 0, indicating no driving automation, to full automation at level 5. In between these extremes are four levels of division of control between human and system. Cars at level 1 and 2 are currently being sold commercially, and require, by SAE definition [3], the human driver to monitor the driving environment. In the next levels, level 3 and 4, the car monitors the driving environment, and the human can be requested to respond to intervene in the driving (also referred to as a transition of control). In level 3 such human intervention is required, whereas in level 4 this is optional and the car needs to have a fallback strategy in case the human does not intervene. At the final level, level 5, human assistance is not needed, but even then the car might occasionally ask for human input (for example, what route to take if there are multiple options). Stated differently, at the upcoming levels of automation (SAE levels 3, 4, and 5), human drivers can be signaled to provide input to the vehicle.

Although there is no specification or standard for how a vehicle might signal that a human’s input is requested, a likely candidate are audio signals. One advantage of audio signals is their near omnidirectional character (i.e., perception of auditory stimuli is "gaze-free") which yields a higher chance of being perceived compared to visual signals. Moreover, auditory signals are already widely used in vehicles for notifications (often supported by visual signals), for example to alert that the vehicle is running out of petrol, as a seatbelt warning, or as proximity indication for parking sensors. Therefore, investigating susceptibility to auditory signals for (semi-) autonomous vehicles is timely, given the pace of development of automated vehicles, in which there is shared control between the human driver and the vehicle.

A potential complication is that the extent of auditory detection and processing may depend on the extent of engagement in an ongoing task, such as driving (for a recent review see [4]). This makes sense from a perceptual-load perspective [5,6], which holds that increasing perceptual demands within an ongoing task reduces the extent of processing of stimuli that are task-irrelevant or that are only of secondary relevance. In such perceptual load experiments, performance indices of load-dependent processing reductions are typically based on interference from the irrelevant stimuli with processing of task-relevant stimuli, or additional task elements such as a posteriori subjective report (see reviews in [4,6]).

Cognitive load can also be measured by exposing participants to a stimulus without requiring a behavioral response [7–9]. By not requiring a behavioral response, the approach is unobtrusive. Instead, measures of human electrocortical activity as available in the electroencephalogram (EEG) can be used. EEG reflects the waxing and waning of postsynaptic potentials that necessarily precede possible action potentials, when this is synchronized across ten thousands of cortical neurons. The common interpretation is that both spontaneous EEG and the change in EEG in response to discrete events can reveal the involvement of specific cognitive processes (for a broader introduction see Luck [10]).

The unobtrusiveness of EEG (i.e., its applicability without additional task demands on part the participant) makes the approach suitable for use in an automotive setting. Note that numerous studies have utilized measures of spontaneous EEG to monitor vigilance in driving or driving-like conditions (e.g., [11–14]). Within the present approach, however, susceptibility to a discrete auditory stimulus, is assessed by deriving from the EEG discrete electrocortical responses (or event-related potentials, ERPs) to these very auditory signals. One fruitful implementation of this principle has been demonstrated by Wester and colleagues [7–9] in a variety of driving(-related) conditions, involving the recording of the frontal P3 or fP3.

The fP3 (also known as P3a or novelty P3) is preferably recorded in response to task-irrelevant unique, relatively unexpected, environmental sounds (“novels”; e.g., [15–20]). A dominant current view is that the frontal P3 provides relatively widespread inhibition throughout the brain or a neural signal that instigates such widespread inhibition (cf. [15,21–23]). As a generic mechanism, it is thought to free sufficient neurocognitive and behavioral resources to meet whatever demands a sudden change in the environment (e.g., an unexpected sudden honk of a truck) might impose ([21], p. 122).

The fP3 to novels is commonly recorded when driving is combined with an auditory “three-stimulus-oddball” novelty/ setup. In a three-stimulus-oddball set-up, a participant hears a series of sounds. The large majority of these sounds are constant tones (also referred to as standard), a small subset are deviant tones of a slightly higher frequency, and a third set contain novel sounds (irrelevant unique, relatively unexpected, environmental sounds) (see also Methods for more details on our implementation).

Within a driving context, Wester and colleagues [7–9] demonstrated that the fP3 component is significantly reduced during driving compared to a stationary control condition. This has also recently been replicated by Scheer, Bülthoff, and Chuang [24]. The interpretation of these results is that the brain is less inclined to meet the demands that a sudden change in the environment might impose when a person is driving compared to when the car is stationary.

A further finding in the Wester et al. studies is that directing attention to auditory information in general restores the driving-reduced fP3 to a considerable extent. In the typical “three-stimulus-oddball” novelty/ fP3 setup, novel stimuli are interspersed (on 10% of the trials) in a sequence of ‘standard’ tones (e.g., 1000 Hz, 80% of all stimuli) and ‘deviant’ tones (e.g., 1100 Hz, also on 10% of the trials). It has turned out that, relative to a driving-only condition in which the oddball stimuli are completely irrelevant, instructing participants to manually respond to the deviant tone enhances the fP3 to the novels [7–9]. This is important because it suggests that susceptibility for rare but salient and potentially relevant audio signals (e.g., alerts to signal a transition of control) can be augmented by having human controllers engage in a second auditory task.

What might these results predict for autonomous driving? One could argue that autonomous driving is comparable to non-autonomous driving given the aspects of moving through space and being exposed to changing visual input that is safety-critical. Alternatively, the autonomous condition may be more comparable to being stationary, in the sense that, unlike manual driving, there is no need to control a vehicle. How does an automated setting then influence a human’s susceptibility to auditory signals? We foresee three possible outcomes. The susceptibility in automated driving can be (1) reduced, similar to the observed reduction in non-autonomous driving, (2) unabated with respect to a non-driving situation, similar to stationary, or (3) lie somewhere in between these extremes.

To investigate how autonomous driving affects human susceptibility to auditory alerts, we conducted an experiment in a driving simulator where participants experience three conditions in a within-subjects manipulation: (1) watching a stationary screenshot of the driving simulator, i.e., a non-driving situation, (2) driving manually in a simulator, and (3) being driven by a simulated autonomous car. Meanwhile, oddball stimuli were presented at a regular pace to allow for measurement of the fP3. In addition, we compared whether there is a difference between a condition where an active response to auditory stimuli is required and a passive condition in which no such response is needed.

## Methods

### Participants

To determine the number of participants, we conducted a power test. Starting point was the effect size that was measured in previous research by Wester et al. [7] on the same metric of interest as we use here (fP3 amplitude, see section on measurement and data analysis). Wester et al. found an effect size (d) of 1.8 for the difference between stationary and regular driving, which we took as input for our power test. We also set our intended level of significance (alpha) at 0.05, and intended power (one-tailed) at 0.8. If this information is entered into G*Power 3.1.9.2, it yields a required sample of n = 4 participants. Because in the present study the autonomous condition was included (i.e., we have a third driving condition; not just stationary and regular driving as in Wester et al.), and a comparison between the passive and the active conditions was envisaged, we anticipated that more participants were needed to detect effects between these 3 x 2 conditions (see section on Design for details on the experimental design). We decided to include at least four times as many participants (i.e., at least 16).

**Fig 1:**
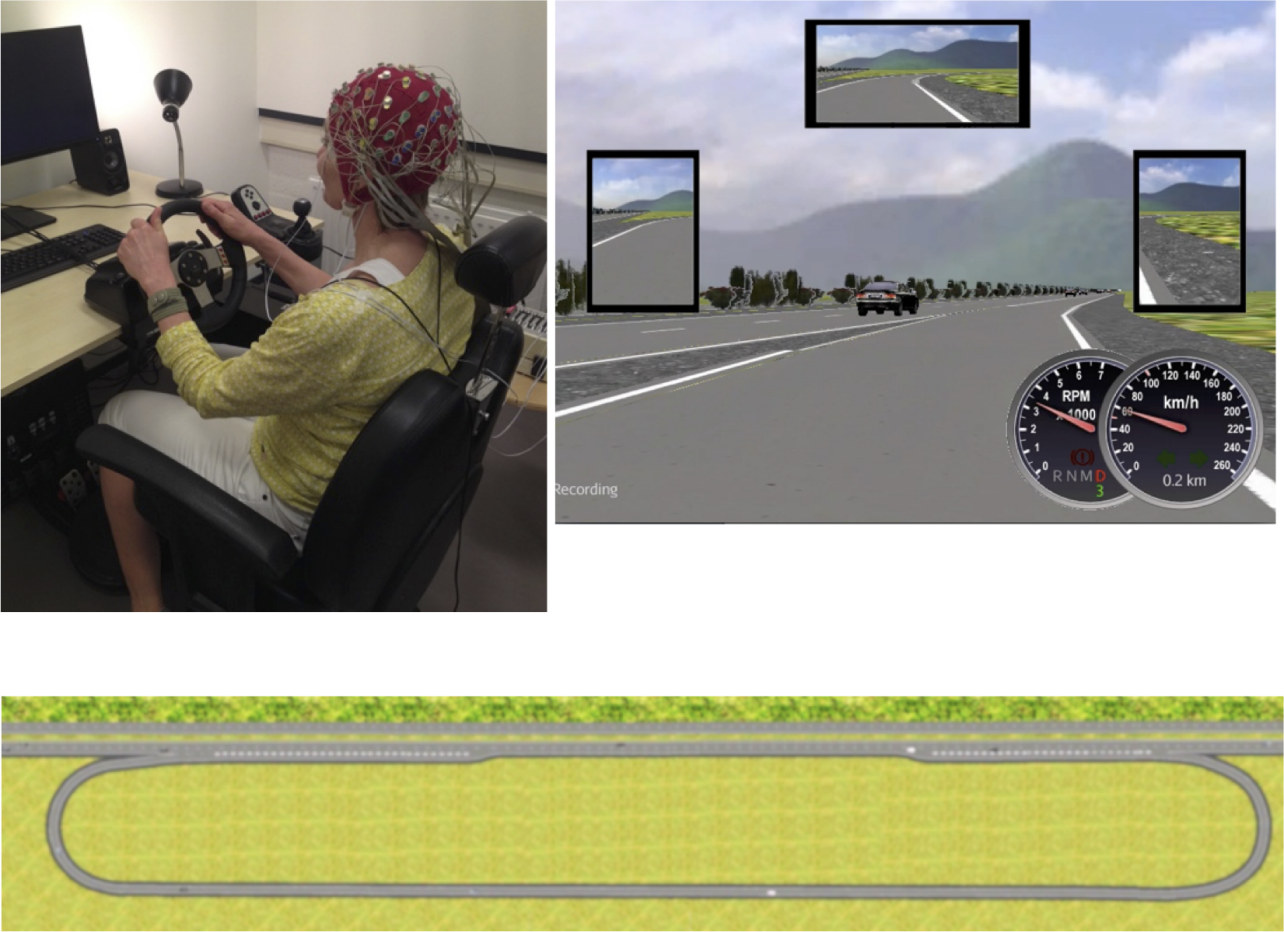
Set-up of the driving study. (Top-left: 1A) Picture of the driving simulator setup with an individual (not a participant) wearing an EEG cap and 64 electrodes attached. The individual has given written informed consent (as outlined in PLOS consent form) to publish these case details (i.e., to publish this picture). (Top-right: 1B) Screenshot of the driver’s perspective on the simulated road. (Bottom: 1C) A schematic view of the course with starting point indicated and single lane road on the bottom and 2 lane highway on the top.

Eventually eighteen participants (7 M; 11 F) were recruited from a student population using opportunity sampling (ages 20 to 25 years old, *M* = 22.06 years, *SD* = 1.39 years). All participants were native Dutch speakers and reported to have normal or corrected-to-normal vision and normal hearing. This study also required the possession of a driver’s license (possession varied from 2 weeks to 6 years, *M* = 3.56 years, *SD* = 1.55 years). One participant (participant 8) did not adhere to task instructions, and showed reckless driving during the experiment. We therefore recruited another participant (19) to replace this participant. The above statistics of participant set are of the final set of eighteen participants (participants 1–7 and 9–19). The experiment was approved by the ethics committee of the Faculty of Social and Behavioral Sciences of Utrecht University (approval number FETC16-042). All participants gave written informed consent prior to the experiment. Fig 1 contains a picture of an individual in the set-up (not a participant). This individual has given written informed consent (as outlined in PLOS consent form) to publish this Figure.

### Materials

#### Driving simulator

We used a low-fidelity desktop-based driving simulator. Participants were seated in an adjustable chair at about 120 cm from the simulator screen. A 29-inch screen showed a road from the driver’s perspective. Three mirrors and analogue speed and rpm indicators were presented on the screen. A Logitech G27 steering wheel and pedals were used to perform the driving task. The simulated car was set to automatic transmission. The set-up is shown in Fig 1A and a screen-shot of the driver’s perspective on the road is shown in Fig 1B.

For the software, we used a modified version of the MotorwayTask in Open Driving Simulator (OpenDS) version 4.0 [25]. This scenario consisted of a loop, as illustrated in Fig 1C, which participants had to drive twice. Participants started on a single-lane road, with a maximum posted speed limit of 70 km/h. Then they entered a two-lane highway via a right-sided ramp. This highway had a maximum posted speed limit of 100 km/h. After driving on the highway for 1000 meters, they had to exit the ramp on the right, to enter the single lane road again. After 1000 meters they were at the start of the scenario again. To increase the impression of driving on a highway, other cars were also present on the road. Each driving trial lasted approximately 5 minutes.

#### Auditory novelty oddball paradigm

Participants were presented with three types of sounds: a standard tone, a deviant tone, and novel stimuli. The standard tone was a 1000 Hz tone, presented for 400 ms. The deviant tone was 1100 Hz tone, presented for 400 ms. The novel stimuli consisted of 100 different environmental sounds retrieved from a database [26], such as animal sounds (e.g., dog barking, bird chirping), human sounds (e.g., coughs, laughs, sneezes), or other sounds (e.g., hammering, water running). Novel sounds varied in duration between 159 ms and 399 ms. In each block, 130 oddball stimuli were presented. Of these oddball stimuli, 80% were standard tones, 10% were deviant target tones, and 10% were novel sounds (cf. [7]). The interval between two stimuli was 2.34 s (measured between onsets of the two sounds). All sounds were played binaurally using Presentation software (Neurobehavioural Systems) at 75 dB through Earlink earphones.

### Design

Autonomous driving was compared to manual driving and stationary/non-driving using a 2 (oddball response requirement) x 3 (driving mode) mixed factorial design. Between subjects we manipulated the oddball response requirement. That is, half of the participants was instructed to press a button on the top left side of the steering wheel as quickly as possible whenever they heard a deviant tone (active condition), the other half of the participants had no button press requirement at all (passive condition).

Within-subjects, the driving modes consisted of three levels: stationary, driving, and autonomous. In the stationary condition, participants were asked to look at a still frame, which showed a screenshot of the simulated road. In the driving condition, participants were asked to drive on a track in a driving simulator (see Fig 1C). In the autonomous condition, participants were given the impression of being driven by a semi-autonomous car by showing them a pre-recorded screen capture video of the track as used in the driving condition (i.e., a pre-recorded screen-recording of one of the experiment leaders driving). To provide the illusion that the autonomous driving condition was indeed a driving condition, the recording also included the startup screens of the simulator, identical to the driving condition. This made it visually indistinguishable from running the actual simulator software. As the driving simulator did not require a driving response from the user, it is ambiguous how to map the autonomous condition directly to a specific level of the SAE framework [3]. Given that the car drives itself (including lane change), the simulation can be considered SAE level 3 or higher.

Each participant performed each driving mode condition four times, creating a total of 12 blocks. The order of blocks (and therefore, conditions) was semi-randomized. Per set of three blocks, all three driving modes (*S*tationary, *A*utonomous, *D*riving) were experienced in the same order (e.g., *S*-*A*-*D*—*S*-*A*-*D—S*-*A*-*D—S*-*A*-*D*). Each ordering was experienced by both a participant in the active condition, and one in the passive condition. Since the total permutations was 6, each unique ordering was experienced by at least one participant in the active condition and one participant in the passive condition, and three orderings were experienced by two participants per active and passive level.

### Procedure

After receiving general instructions and walking through the consent procedure, participants started by practicing the driving task. After instructions and adjustment of the simulator seat, participants either were driven autonomously, or drove themselves for one block (order was balanced between participants). The driving practice was followed by an oddball practice. For the oddball practice trials, the oddball task was shortened from 130 to 42 stimuli but the proportion of novel and deviant tones was kept in accordance with the experimental trials (i.e., 80-10-10%). After the practice trials, we checked verbally whether no complaints due to motion sickness occurred. None occurred during the experiment.

After the practice trials, the EEG cap with electrodes was applied. Participants then completed the twelve experimental blocks (see section on design). Between blocks was a small break (roughly 1 minute), and after six blocks a slightly longer break (i.e., about 5 minutes). The aim of the break was to not overstrain the participant. They were asked to stay in their seat and relax. No specific instructions were given on what to do during the break.

For purposes of internship training, after the last block of each driving condition (blocks 10, 11, and 12), we asked participants to fill out a NASA raw-TLX questionnaire [27]. The results of this questionnaire are not reported here.

After the experimental trials, the EEG cap and electrodes were removed. Participants were then requested to complete a questionnaire that collected demographic data and their subjective experience of the experiment. For purposes of internship training, a questionnaire on impulsivity was also administered. The results of this questionnaire are not reported here. The total procedure took between 120 and 150 minutes, mostly depending on the length of breaks and time needed to fill-out the questionnaire.

### Instructions to participants

Participants received various explicit instructions verbally, and later also on the screen of the simulator. For the oddball task, participants in the active response condition were instructed to respond as fast as possible to the deviant tone and that no response was needed to other auditory stimuli. Participants in the passive condition were instructed to respond to none of the auditory stimuli.

With respect to the driving task, participants were told to follow the road and complete the motorway task as described in the materials section. Furthermore, participants were asked to adhere to normal traffic regulations and to drive safely. For the autonomous and stationary conditions, participants were told to stay focused on the screen. In all conditions, participants were instructed to keep their hands on the steering wheel.

### Measurement and data analysis

#### ERP recording and analysis

To be able to measure the susceptibility of the brain to auditory stimuli, event-related potentials (ERP) in response to the oddball stimuli were measured using electroencephalogram (EEG) recordings. EEG was recorded by a BioSemi ActiveTwo system at 2048 Hz. 64 active Ag-AgCl electrodes were positioned according to the international 10/10 system [28]. Electrodes were also placed on left and right mastoids for purposes of offline re-referencing. To be able to compensate for eye-activity related noise, we recorded horizontal and vertical eye movements and eye blinks, for which electro-oculography (EOG) electrodes were placed on both outer canthi of the eyes as well as above and below the right eye in line with the pupil.

After the experiment, ERPs were analyzed using Brain Vision Analyzer 2 software (Brain Products GmbH, München, Germany). Next to a 50 Hz notch filter to compensate for noise from the mains, 0.16 Hz high-pass filter with a slope of 24 dB/oct and a 30Hz low-pass filter with a slope of 24 dB/oct were applied to the EEG data. Data were referenced to the average of left and right mastoid signal. Trials containing false alarms (i.e., button-press responses to the standard or novel stimuli), misses (i.e., failed responses to the deviant target tone), and invalid responses (i.e., responses outside the 100–950 ms interval, after correction for a delay—see below) were removed.

After these initial steps, the data were binned per condition (stationary, autonomous, and driving) and per oddball stimulus (standard, deviant, and novel), resulting in 9 bins. To control for eye movements and blinks, an ocular correction was applied to every segment using the Gratton, Coles and Donchin [29] method. The data were baseline corrected over the 100ms interval preceding the stimulus onset. Subsequently, artifacts were automatically rejected (when at least one of the following conditions was met within the epoch: maximum voltage step > 120 μv/ms; maximum difference > 100 μv; minimum activity < 0.5 μv). Average ERP waveforms over all participants were calculated per stimulus type (standard, deviant, and novel) per condition (driving, stationary, and autonomous).

Difference waves were calculated by subtracting the average ERP for the standard tones from the average ERPs for the novels for each driving condition. Based on Wester et al. [7], we analyzed the mean of the difference wave at electrode FCz in the interval 325–375 ms after stimulus onset. During the analysis of the experiment, we became aware, and cross-tested, that the audio presentation was systematically delayed by 50 ms compared to the logged time. We therefore corrected the reported time log, such that the ERP response wave is relative to actual time presentation, not the logged time (i.e., 50 ms later than logged). In our analysis of the invalid responses (discussed above) we therefore also use an adjusted interval of 100 to 950 ms (compared to 150 to 1000 ms in [7]).

#### Reaction time (active response condition)

For the active condition only, we could measure how quickly participants responded to a deviant tone. Specifically, we analyzed the time interval between the onset of the deviant tone, and the onset of the button press on the steering wheel as a measure of reaction time. Button presses were registered by the simulator computer and transferred to the BioSemi as marker codes directly. Reaction times were also corrected by subtracting 50 ms to account for the delayed stimulus presentation.

#### General questionnaire

The questionnaire data was used to check for inconsistencies (e.g., whether participants had not understood instructions). One participant was removed based on this description (see participants section). No other obvious inconsistencies were found, therefore not providing grounds to a priori remove participants from the analysis.

#### Statistical analysis

Statistical analyses were conducted in SPSS 24. We used a 2 x 3 mixed multivariate-contrast ANOVA, with Oddball response requirement as a between-subjects factor (two levels: passive and active) and Driving mode as within-subjects factor (three levels: stationary, autonomous, and driving, transformed to linear and quadratic contrasts). Throughout all analyses, a significance level of α = .05 was used. For significant effects, the *d* effect size was calculated as the square root of the *F*-value divided by the square root of the sample size.

Given omnibus effects of driving mode, we used pairwise contrast tests for differences between the three driving conditions. In the introduction, we anticipated three possible outcomes. If the fP3 component is reduced in a similar fashion in the autonomous condition as it is during driving, this would be reflected in differences between autonomous and driving on the one hand, and stationary on the other. If the fP3 component is unabated in the autonomous relative to the stationary condition, there would be significant differences with the driving condition only. If the magnitude of the fP3 in the autonomous condition lies somewhere in between the stationary and the regular driving condition, this would manifest in a significant difference between these latter two condition, with smaller differences between either of these conditions and the autonomous condition.

## Results

### Susceptibility to novel sounds (fP3)

**Fig 2:**
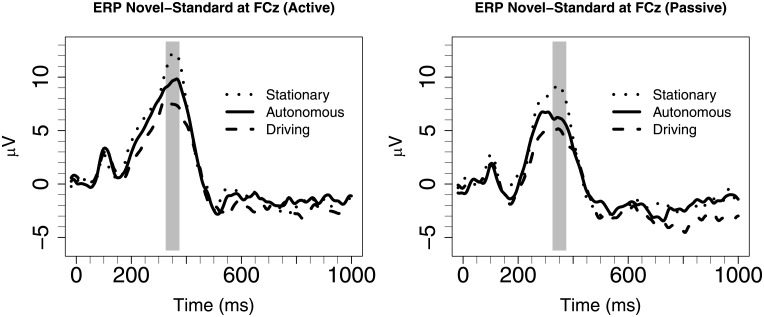
Grand-average difference ERPs (novel minus standard) (microvolts) in different driving modes (lines), relative to onset of auditory stimulus (0 s) for the active condition (left panel) and the passive condition (right panel). The shaded area indicates the epoch of frontal P3. Both autonomous (solid line) and driving (dashed line) show a reduced frontal P3 peak compared to stationary (dotted line).

Driving mode had a significant main effect on fP3 amplitude, *F*(2, 15) = 12.4, *p* < .005. The difference waves of the three driving conditions are shown in Fig 2 for the active condition (left panel) and passive condition (right panel). The fP3 was strongest in stationary (*M* = 10.3 μV, *SD* = 3.5 μV), followed by autonomous (*M* = 7.8 μV, *SD* = 3.4 μV), followed by driving (*M* = 6.2 μV, SD = 2.9 μV). Hence, the reduction in fP3 in autonomous conditions was in between that observed in stationary and regular driving (i.e., as in option 3 from the introduction). Pairwise comparisons yielded significant differences between stationary and driving (*p* < .001, d = 1.21), between autonomous and driving (*p* = .048, d = 0.51), and between autonomous and stationary (*p* = .008, d = 0.71). Hence, the effect size for stationary versus driving was somewhat smaller than in the Wester et al. (2008) study (d = 1.8), but still in the large range. The effects sizes for the comparisons involving the autonomous condition, although significant, were clearly smaller (than 1), confirming the pattern specified in option 3.

**Fig 3:**
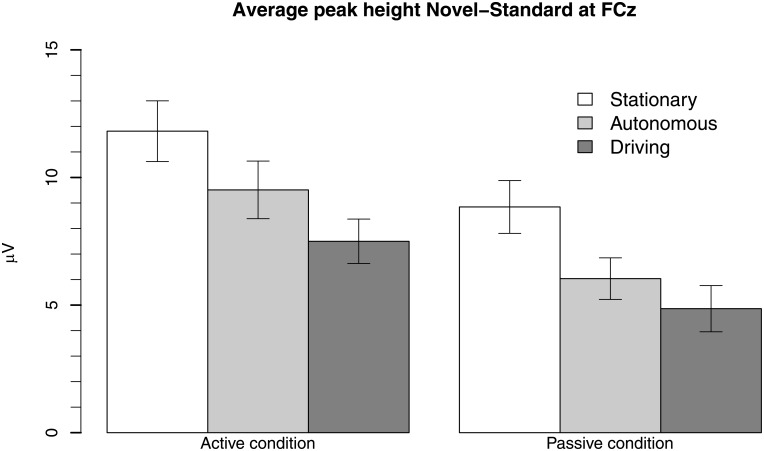
Bar plot of average fP3 response per oddball response category and per driving condition. The data show a main effect of oddball response category, and a main effect of driving conditions.

There was also a main effect of oddball response requirement on fP3 amplitude. The active response requirement group had a significantly larger fP3 (*M* = 9.6 μV, *SD* = 3.6 μV) compared to the passive group (*M* = 6.6 μV, *SD* = 3.2 μV), *F*(1, 16) = 8.00, *p* = .01, d = 0.67. There was no significant interaction between the driving mode and Oddball response requirement, *p* > .1. Fig 3 shows a bar plot of the average fP3 response in the active and passive condition for each of the three driving conditions. The Figure illustrates the main effect of response requirement (that active conditions generated higher fP3 amplitude compared to the passive conditions) and the main effect of driving condition (i.e., that fP3 amplitudes are highest in the stationary condition, followed by the autonomous condition, followed by the driving condition).

**Fig 4:**
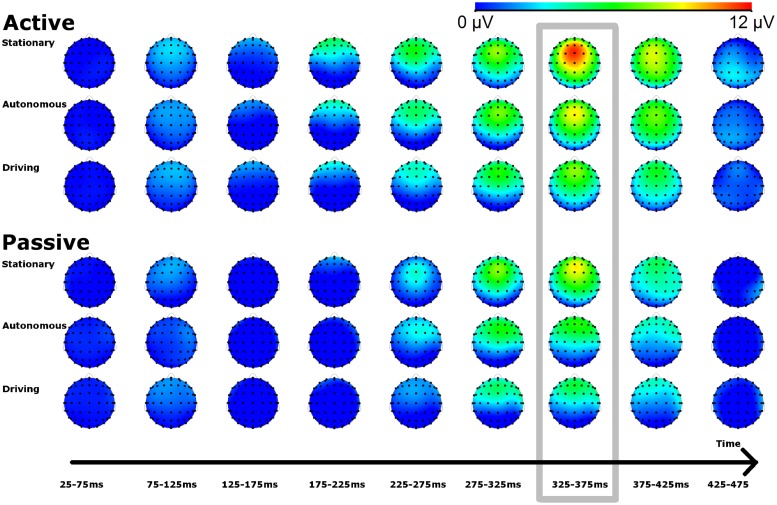
Scalp distribution of difference in EEG activity in response to the novel relative to the standard. fP3 activity is strongest around the FCz electrode (4^th^ from the nose down) in the 325-375ms interval.

To gain further insight in the wider cortical response to the novel stimuli, Fig 4 plots a heatmap of the distribution of amplitude (μV) across the scalp (i.e., all 64 electrodes). Amplitudes concern the difference wave between the novel and standard ERP (i.e., similar to Fig 2). The data were based on measurements at all 64 electrodes and binned in bins of 50 ms, starting at 25 ms after stimulus onset. Values were capped at 0 μV and 12 μV (i.e., values outside this range are given the color of the boundary). Dots show the locations of the 64 electrodes in their default reference position. The FCz electrode is the fourth electrode from the nose down. The top three rows are data from the active response condition for stationary, autonomous, and driving. The bottom three rows are the data from the corresponding driving condition in the passive response condition.

Fig 4 again highlights that the fP3 activity is strongest in the 325–375 ms interval (7^th^ scalp picture from the left; surrounded by a grey border for emphasis). The Figure also illustrates that the amplitude is the highest surrounding the FCz electrode. Moreover, the Figure also illustrates that fP3 activity is reduced in the passive response condition compared to the active response condition, and that the fP3 activity is reduced in autonomous compared to stationary, and in driving compared to autonomous.

### Oddball response time (RT)

In the active condition, we measured the response time between the moment a deviant sound was presented and the moment a button was pressed. The MANOVA revealed no significant effect of driving condition (*p* = .127). Looking at the means, reaction time was highest in the driving condition (*M* = 594 ms, *SD* = 107 ms), followed by the autonomous condition (*M* = 551 ms, *SD* = 108 ms), followed by the stationary condition (*M* = 545 ms, *SD* = 98 ms).

We also analyzed response time variability, through analysis of the standard deviation of reaction time in response requirement. The MANOVA again revealed no significant effect of driving condition (*p* = .122). Looking at the means, standard deviation was highest in the driving condition (*M* = 140 ms, *SD* = 25 ms), followed by the stationary condition (*M* = 127 ms, *SD* = 32 ms), and the autonomous condition (*M* = 116 ms, *SD* = 24 ms).

In previous studies, different patterns were found for both measures: one study found that response time to the deviant tones were more variable (but not longer) during simulated driving [7], whereas another found that mean reaction times were longer (but not more variable) during on-the-road driving [9]. It should be noted that our reaction time analysis was based on only the 9 subjects in the active condition. Observed power to detect any reaction time differences (for mean reaction time) between driving conditions as reported by SPSS was only 0.384. This may be contrasted to the observed power to detect driving-condition effects on fP3 across the two groups, which was 0.985. To attain more information on the likelihood of not rejecting H0 while H1 was actually true, an additional Bayesian analysis was conducted using JASP [30]. For the analysis of mean reaction times, the analysis revealed a Bayes factor of 2.6, implicating that the alternative hypothesis (i.e., that driving condition has an effect on mean reaction time) is 2.6 times more likely compared to the H0 hypothesis (i.e., that driving condition has no effect on response time). A Bayes Factor between 1 and 3 is seen as anecdotal evidence ([31], see also Chapter 7 in [32]). A subsequent Bayesian post-hoc test revealed Bayes factors of 2.3 for stationary versus driving, 1.4 for autonomous versus driving, and 0.4 for autonomous versus stationary. Note that Bayes factors smaller than 2 indicate very low likelihood of rejecting the H0 even with substantial increase in power. Hence, the current analysis suggests that with higher power, a significant omnibus effect of driving condition on reaction time may still be observed, but that this would be entirely due to the difference between stationary and driving; apparently, differences between autonomous and the other conditions are not sufficiently robust across participants (and for the difference between autonomous and stationary it is 1 / 0.4 = 2.2 times more likely that the null hypothesis is true).

When we performed a similar Bayesian analysis for response time variability, we found a Bayes factor of 0.949, suggesting that our data provides no conclusive evidence of whether driving condition has an effect on response time variability (i.e., the H1 and the H0 have a comparable likelihood based on the current data).

## Discussion

We investigated susceptibility to auditory signals during autonomous driving, compared to regular driving and being stationary. We recorded the frontal P3 (fP3) ERP (Event-Related Potential) component as a metric of susceptibility [7–9,21]. Consistent with this earlier work, we found that fP3 was reduced when participants were driving compared to when they were stationary, suggesting that susceptibility to novel stimuli is reduced when driving [21]. The novel finding is that under autonomous driving conditions the fP3 is also reduced compared to stationary (cf. [7]).

In our introduction, we mentioned three potential outcomes of our study: The susceptibility in automated driving can be (1) reduced, similar to the observed reduction in non-autonomous driving, (2) unabated with respect to a non-driving situation, similar to stationary, or (3) lie somewhere in between these extremes as autonomous driving has characteristics of both driving and non-driving. Taken together, our results rule out alternative 2 and lend most support for alternative 3: there was a significant difference between stationary and autonomous condition, but the effect size of this effect was smaller compared to the difference between regular driving and stationary.

The results of our study also suggest that susceptibility to auditory stimuli is in general better (i.e., frontal P3 components with a larger amplitude) in cases where participants are required to actively respond to some of the task (active condition) compared to when they do not need to respond to such a signal. This improvement was measured as a main effect, and therefore holds for being stationary, when being driving autonomously, and when driving oneself.

The reduced susceptibility during both regular driving and autonomous driving is consistent with the perceptual-load perspective [4–6], implying that being sufficiently engaged in an on-going task (i.e., driving yourself, or being driven) reduces the extent of processing of task-irrelevant stimuli. As in the Wester studies [7–9], we also found that fP3-indexed susceptibility was enhanced again by making auditory information relevant to a task as well (specifically, fP3 to novels was enhanced by having subjects actively indicating the detection of non-novel target deviants). This is potentially important, as it suggests that susceptibility for very infrequent alerting signals can be enhanced by adding (in a real driving situation) a more or less continuous auditory task. As reported in [7,9], introduction of the (active) oddball task has no effect on aspects of basic vehicle control (weaving and speed). Future research must reveal whether this also holds for the tactical and strategical aspects of driving, which become more important in cases where a (semi-) autonomous vehicle takes over mostly the basic vehicle control (e.g., adaptive cruise control and maintaining lateral position in SAE level-2 automation).

The difference between our active and passive conditions may also be compared to that between SAE level 3 of automation in which a response is required (comparable to our active condition), and SAE level 4 in which a response to an alert is optional (comparable to our passive condition). Our results suggest that susceptibility to SAE level 4 alerts may be reduced (cf. our results in the passive condition). Therefore, even though auditory alerts might be provided in a SAE level 4 automated vehicle, their passive nature might make them not that effective. Further empirical research is needed to test this prediction.

It is an empirical question what underlies the better susceptibility in the active condition compared to the passive condition. Possibly, performing a task with respect to a specific auditory event (the deviant target) yields a higher prioritization for auditory information in general (including the novels). In turn, this results in allocation of apparently still free processing capacity (manifest in fP3), without interfering with basic vehicle control. A further possibility is that engaging in an additional task reduces underload [33]. More data and theory are needed to disentangle these hypotheses.

In the active condition, reaction times to the deviant target were shortest in the stationary condition, followed by autonomous, followed by regular driving. However, our MANOVA revealed no main effect, and a Bayesian analysis revealed that although the trend is that there might be a significant effect of driving condition on reaction time, the evidence is not enough to draw solid conclusions. We also did not find a significant effect of driving condition on response time variability.

These results differ slightly from the findings by Wester and colleagues. In their studies, manual response time to the deviant tones were more variable (but not longer) during simulated driving [7] and longer (but not more variable) during on-the-road driving [9].

Pulling it all together, it appears that adding a more or less continuous auditory task increases fP3, while fP3 is still smaller during autonomous driving than during stationary. At the same time, within that task, participants show no appreciable decline performance from the stationary to the autonomous conditions. This can be viewed as a dissociation between on the one hand, the ability to detect potentially relevant auditory information (the novels), and on the other, the ability to react to unambiguously relevant auditory information (the deviant targets). Consistently, based on target-elicited ERP results, Wester ([9], p. 133) concluded that there may have been “an increased investment of effort to compensate for the increased task-load imposed on the oddball task during driving compared to non-driving.”

### Implications for practice

Regarding the implications for practice, our results provide four reasons for some caution in the use of auditory alerts in semi-autonomous vehicles as a cue for handover (also referred to as a transition of control). First, the susceptibility in the autonomous condition was significantly lower compared to the stationary condition, thereby suggesting that people’s susceptibility to audio signals is reduced. The implication is that there may be a need for more research and development with respect to the design of alerts. For example, in our own work, we have demonstrated how early warnings can be beneficial to allow for a smooth handover between vehicle and driver in level 3 automation [34].

Second, adding an auditory task seems to enhance susceptibility to some audio signals, but might negatively affect reaction speed (i.e., if the trend that our Bayesian analysis detected persists in future studies). This dissociation needs to be resolved before the current results can be applied in real-life contexts.

Third, our study took place in a controlled setting, where there was only the driving task and the oddball task. However, in their own vehicles, people might do other tasks than driving. More specifically, drivers distract themselves with various non-driving related tasks while driving [35,36], as they do in other non-driving settings [37]. Moreover, a recent literature review suggests that as automation of the vehicle increases, people perform non-driving tasks even more frequently, which results in less awareness of the driving environment, and slower response times [38]. It is an empirical question whether the susceptibility to audio signals is reduced under conditions of distraction while driving. We plan to study the impact of multitasking on susceptibility.

Fourth, and finally, as argued above, our results suggest that susceptibility to SAE level 4 auditory alerts may be reduced. Therefore, designers might reconsider whether such an alert is effective at all. Not using (auditory) alerts seems a too bold conclusion based on our data. However, at the least, our data suggests that careful testing of these alerts is necessary before they are used in actual cars.

### Limitations and future work

Our study was conducted in a driving simulator and therefore suffers from the known limitations of such simulators such as that participants perhaps take more risks compared to when they drive in everyday traffic outside. Nonetheless, previous work by Wester ([9], Chapter 6), has demonstrated that at least in conventional driving, reductions in fP3 that are observed in the simulator setting were also present when measured on the road.

We have studied susceptibility to auditory alerts in a setting without additional distractions. However, given the prevalence of in-car distractions [35,36], including in more automated systems [38], we plan to also study how susceptibility changes when the driver can be distracted. These issues add to other important aspects of driver safety and experience (e.g., [39,40]) including how to avoid confusion about responsibilities in vehicle automation (e.g., [41]), and how to drive efficient and sustainable (e.g., [42]).

With the new gained basic understanding of auditory alert processing, other work can also investigate how the exact type of audio signal impacts susceptibility, which is a large effort given the variety of auditory alerts for in-car settings (for a review of alert types see [43]). Moreover, future work can look at alerts in other modalities such as visual (e.g., see [44] for an example of last-minute visual alerts) and of multimodal signals (e.g., [45]). Finally, the general limitations of signal processing can be investigated, including the impact of competing tones and of false alarms on susceptibility and reaction time (e.g., [46–48]). In the end, providing a signal is not a guarantee that the human acts on it.

## Conclusion

Human susceptibility to auditory signals, as measured by the fP3 (frontal P3), is reduced when driving, but also when being driven autonomously, as compared to a stationary situation. As all levels of autonomous driving, except full autonomy, have some level of shared control between the human and the system, where (auditory) signals might play a role, it is important to consider the limitations of the human brain in terms of susceptibility to auditory alerts. One possible venue to enhancing susceptibility to infrequent auditory events (e.g., handover alerts) lies in the implementation of a suitable additional auditory task in the driving situation.

## Supporting information

S1 File Data files. The zip-file contains the data files that were used in the analysis reported in this manuscript. Within the zip-file a readme file explains what files are included.
(ZIP)
