# Supplementary material for: Susceptibility to audio signals during autonomous driving
Source: PLoS One. 2018 Aug 13;13(8):e0201963. doi: 10.1371/journal.pone.0201963 (PMC6089411; doi:10.1371/journal.pone.0201963)
Supplement: S1 File — The zip-file contains the data files that were used in the analysis reported in this manuscript. Within the zip-file a readme file explains what files are included. (ZIP) [file pone.0201963.s001.zip › readMe.rtf]

Readme belonging to supplementary filesThis zip-file contains various files, listed below.Filename: analysisScript_v20180319_forAppendix.RA R script file that can be opened in R to analyze the data. Contains further information on various steps of the analysis.Filename: ERP_Data.RdataA file that can be read in R (note that the above R script file already loads this data). Note that this is the data after preprocessing. For the raw data, please contact the authors.Filename: reactionTimeSummary_SPSSformat.csvThis file contains summary data on reaction times for each participant in the active response condition.The data is sorted in rows per participant (column PP). For each of the three driving conditions there are three columns:- mean response times (e.g., meanRTAuto is the mean response time in autonomous condition)- standard deviation of response time (e.g., SDAuto is the mean response time for the autonomous condition)- number of observations that were used in the analysis. That is, how many valid measurements were there? (e.g., nrObsAuto contains the file for the autonomous condition)Filename: summaryLogFilesActiveParticipants.csvContains event logs of various responses by the participants that can be used to calculate the means and SDs as reported in the above mentioned file. The R script above provides code to distill the relevant data out of it.
